# Immunological overlap stratification in anti-GBM disease: prognostic differences and serological correlations—a single-center retrospective cohort study

**DOI:** 10.3389/fimmu.2026.1836513

**Published:** 2026-07-07

**Authors:** Yujun Qian, Yuyou Ye, Qian Zhou, Suyan Duan, Chengning Zhang, Yifei Ge, Yanggang Yuan, Changying Xing, Huijuan Mao, Bo Zhang

**Affiliations:** Department of Nephrology, The First Affiliated Hospital with Nanjing Medical University, Jiangsu Province Hospital, Nanjing, China

**Keywords:** anti-glomerular basement membrane disease, anti-neutrophil cytoplasmic antibodies, glomerulonephritis, immunological overlap, patient survival

## Abstract

**Background:**

Anti-glomerular basement membrane (anti-GBM) disease is not a distinct, isolated entity but rather a condition that frequently overlaps with other autoimmune serological profiles. Most notably, it can coexist with antineutrophil cytoplasmic antibodies (ANCA), alongside a diverse spectrum of additional autoantibodies. Despite the occurrence of such antibody overlap in a subset of patients, their specific clinical manifestations and prognostic implications remain poorly defined and largely elusive.

**Methods:**

This study retrospectively enrolled patients diagnosed with anti-GBM disease at the First Affiliated Hospital with Nanjing Medical University between January 2017 and January 2024. Based on serological profiles, enrolled patients were stratified into three cohorts: the classic anti-GBM group (anti-GBM antibodies positive alone), the ANCA-anti-GBM double-positive group, and the anti-GBM-non-ANCA autoantibody overlap group. Clinical, pathological, treatment, and outcome characteristics were systematically compared across the three groups. Additionally, landmark analysis was conducted to assess prognostic discrepancies across distinct time intervals.

**Results:**

A total of 67 patients with anti-GBM disease were enrolled in this study, with 30, 18, and 19 cases allocated to the classic anti-GBM group, ANCA-anti-GBM double-positive group, and anti-GBM-non-ANCA autoantibody overlap group, respectively. The ANCA-anti-GBM double-positive group presented with the highest mean age at onset among the three cohorts. A statistically significant difference was detected in baseline anti-GBM antibody titers across the groups (median: 141.20 vs. 104.25 vs. 181.70; interquartile range: 102.90–257.70 vs. 52.15–137.07 vs. 121.05–555.40; p = 0.044). By contrast, no significant differences were noted in terms of other clinical characteristics, renal pathological findings, and therapeutic regimens. Although the overall survival rates showed no statistical discrepancy among the three groups, distinct trends were observed in early survival outcomes: the ANCA-anti-GBM double-positive group had the poorest early survival, followed by the anti-GBM-non-ANCA autoantibody overlap group, while the classic anti-GBM group exhibited the most favorable early survival profile.

**Conclusion:**

This study identifies the distinct phenotypic traits of anti-GBM disease with concurrent other autoimmune antibodies, underscoring that sole reliance on anti-GBM antibody titers is inappropriate for prognostic judgment and treatment decision-making. For patients with anti-GBM disease overlapping with non-ANCA autoantibodies, intensive treatment regimens may be justified to achieve more favorable clinical prognosis.

## 1 Introduction

Anti-glomerular basement membrane (anti-GBM) disease is a rapidly progressive autoimmune disorder mediated by anti-GBM antibodies targeting the noncollagenous domain 1 (NC1) of the α3 chain of type IV collagen (α3[IV]) (1, 2). The disease predominantly affects the kidneys, typically presenting as rapidly progressive glomerulonephritis syndrome that can progress swiftly to renal failure and may be accompanied by the pulmonary involvement characterized by alveolar hemorrhage. The classic clinical and pathological hallmarks of anti-GBM disease include two core features (1): positive serological testing for anti-GBM antibodies (2); renal histopathological findings under light microscopy showing extensive crescent formation and fibrinoid necrosis of glomerular capillary loops, frequently associated with Bowman’s capsule rupture, coupled with linear deposition of immunoglobulin G (IgG) along the glomerular basement membrane observed on immunofluorescence staining (3).

However, clinical studies have also identified a subset of “atypical anti-GBM disease”, which is defined by linear IgG deposition along the glomerular basement membrane on renal biopsy in the absence of detectable circulating anti-GBM antibodies (4). Compared with classic anti-GBM disease, patients with this atypical variant exhibit milder renal involvement-characterized by reduced hematuria and proteinuria, as well as relatively preserved renal function and rarely develop pulmonary hemorrhage (5, 6).

With the deepening of research into anti-GBM disease, an increasing number of cases have been identified that neither fit the traditional diagnostic criteria nor conform to the previous described features of atypical anti-GBM disease, but instead exhibit distinctive clinical phenotypes. Within the broader anti-GBM disease spectrum (encompassing both classic and atypical forms), 10–50% of patients are found to be seropositive for antineutrophil cytoplasmic antibodies (ANCA), with anti-MPO-ANCA being the most prevalent subtype (7–9). Several rare case reports have described anti-GBM disease accompanied by elevated serum IgG4 levels, with typically severe clinical manifestations and a poor prognosis (10, 11).There were also some cases with co-expression of anti-phospholipase A2 receptor (anti-PLA2R) antibodies have also been documented (12). Furthermore, anti-GBM disease may overlap with other immune-mediated disorders such as immunoglobulin A (IgA) nephropathy or systemic lupus erythematosus; these overlap syndromes are associated with therapeutic responses and clinical outcomes that differ significantly from those of either condition in isolation (13, 14).

To address the coexistence of multiple autoantibodies, we first clarify that this phenomenon reflects an “immunological overlap”—a state in which distinct effector pathways (e.g., neutrophil activation, classical complement cascade, and podocyte antigen targeting) are concurrently activated in a single patient, thereby driving more complex clinical phenotypes and heterogeneous treatment responses.

Building on this mechanistic framework, we propose the novel term “overlap-immune anti-GBM syndrome” to categorize cases with such immunological overlap. Crucially, this syndrome is not defined as a distinct disease entity, but rather as a clinical-serological spectrum, characterized immunologically by the presence of multiple different autoantibodies. This classification distinguishes it from existing “atypical anti-GBM disease” (which focuses on discordance between serology and histology) by centering on the diversity of concurrent autoantibodies and their associated immunopathogenic pathways—filling a gap in current disease stratification.

## 2 Methods

### 2.1 Research design and patient grouping

**Figure 1:**
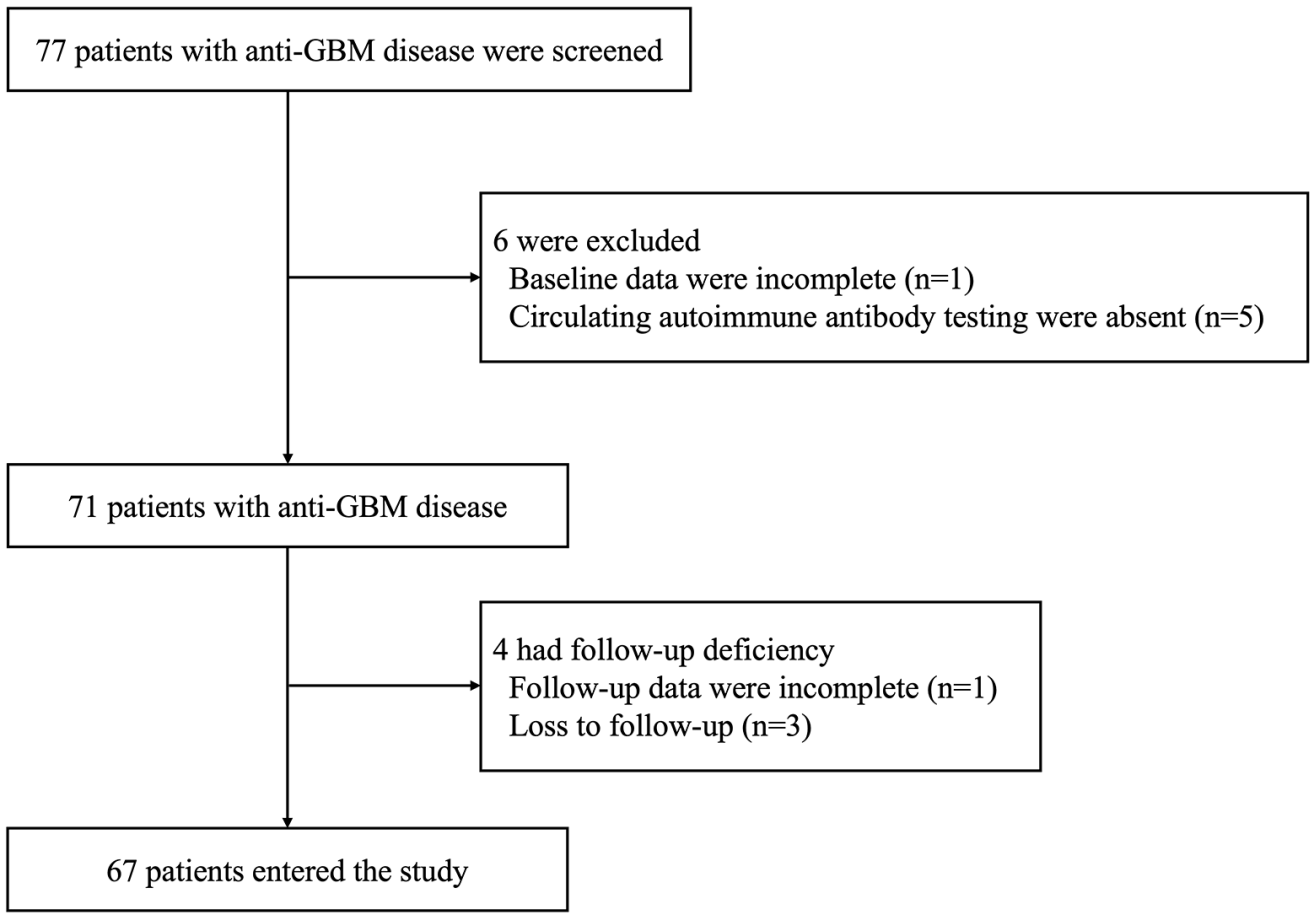
Flow chart of participant selection.

This retrospective cohort study was conducted at the First Affiliated Hospital with Nanjing Medical University, enrolling patients diagnosed with anti-GBM disease between January 2017 to January 2024. All cases were defined by either (i) the presence of circulating anti-GBM antibodies in association with clinical manifestations of alveolar hemorrhage and/or rapidly progressive glomerulonephritis, or (ii) biopsy-proven crescentic glomerulonephritis with linear deposition of IgG along the GBM in the absence of another attributable cause (such as diabetes mellitus or paraproteinaemia). Patients with incomplete clinical, pathological, or follow-up data were excluded from the analysis. Given that certain diseases, such as infective endocarditis, brucellosis, tuberculosis, and other chronic inflammatory infections, may generate secondary autoantibodies through molecular mimicry and polyclonal immune activation, the patients with anti-GBM disease enrolled in this study underwent corresponding examinations, including blood culture, etiological examination, and echocardiographic evaluation, to exclude the aforementioned suspected infections. The patient enrollment flowchart is presented in **Figure 1**. The study protocol was reviewed and approved by the Institutional Ethics Committee of the First Affiliated Hospital with Nanjing Medical University, Nanjing, China. Written informed consent was obtained from all enrolled participants or their legal representatives prior to data collection.

Enrolled patients were stratified into three distinct subgroups based on their circulating autoantibody profiles, to align with the “overlap-immune anti-GBM syndrome” framework:

- Classic anti-GBM group (Group 1): Defined by exclusive positivity for anti-GBM antibodies (no other detectable autoantibodies).
- Anti-GBM-ANCA double-positive group (Group 2): Patients concurrently positive for both anti-GBM antibodies and antineutrophil cytoplasmic antibodies (ANCA).
- Anti-GBM-non-ANCA autoantibody overlap group (Group 3): Patients positive for anti-GBM antibodies plus other serological autoantibodies (ANCA excluded) (**Table 1**).

**Table 1:** Antibody repertoire of the anti-GBM-non-ANCA autoantibody overlap group.

Case	GBM	ACL	PLA2R	dsDNA	ANA	Jo-1	SSA	Ro-52	SSB	Sm	U1-RNP
1	+								+		
2	+							+			
3	+			+	+		+				+
4	+			+							
5	+				+					+	+
6	+				+		+				+
7	+				+		+	+			
8	+	+									
9	+	+									
10	+	+				+					
11	+	+									
12	+	+									
13	+	+			+						
14	+	+			+						
15	+	+									
16	+		+								
17	+		+		+						
18	+	+	+								
19	+	+	+								

GBM, anti– glomerular basement membrane antibody; ACL, anti-cardiolipin antibody; PLA2R, anti-phospholipase A_2_ receptor antibody; dsDNA, anti– double-stranded DNA antibody; ANA, anti-nuclear antibody; Jo-1, anti-histidyl-tRNA synthetase antibody; SSA, anti-Sjögren-syndrome-related antigen A antibody (Ro); SSB, anti-Sjögren-syndrome-related antigen B antibody (La); Ro-52, anti-Ro-52 (TRIM21) antibody; Sm, anti-Smith antibody; U1-RNP, anti-U1 small nuclear ribonucleoprotein antibody.

### 2.2 Data collection and follow-up

At the time of enrollment, we collected comprehensive baseline data from all patients, including demographic characteristics, clinical manifestations, laboratory test results, and renal biopsy pathological findings. The enrolled patients were followed up prospectively from the date of diagnosis until January 2024. Specifically, we documented:

- Demographic and clinical variables: Sex, age, smoking history, preceding infection, pulmonary involvement, gross hematuria, oliguria, anuria and blood pressure.
- Laboratory parameters: Complete blood count, serum creatinine, urea, total protein, albumin, globulin, total cholesterol, liver enzyme levels, C reactive protein, and urinary findings; autoantibody titers (anti-GBM, ANCA, antinuclear antibodies [ANA], anti-double-stranded DNA [anti-dsDNA], anti-cardiolipin [ACL], anti-ENA profile [SSA/Ro, SSB/La, Smith, RNP, Scl-70, Jo-1, etc.], and anti-PLA2R) were measured by ELISA.
- Renal biopsy pathology data: Deposition patterns of immunoglobulin, complement, and fibrinogen, histomorphological features, as well as indicates of acute and chronic renal lesions.
- Follow-up outcomes: Treatment regimens administered, all-cause mortality, and renal survival status throughout the follow-up period. The primary endpoint of the study was defined as all-cause mortality. Overall survival (OS): Defined as the time from the date of anti-GBM disease diagnosis to the occurrence of all-cause mortality (the primary endpoint event); patients without mortality by the follow-up cutoff (January 2024) were censored at the last follow-up date. Early survival: Referring to the survival status within the period from diagnosis to the landmark time point (600 days post-diagnosis), with the same endpoint event (all-cause mortality) as OS.
- Follow-up and Data Management: Data collection frequency: Patients were followed up every 1–3 months in the first year post-diagnosis, and every 6 months thereafter; survival status was confirmed via outpatient visits, telephone follow-up, or medical record review.

### 2.3 Statistical analyses

We first used the Kolmogorov-Smirnov test to evaluate the normality of continuous variables. Normally distributed continuous data were presented as the mean ± standard deviation, while skewed data were expressed as median (interquartile ranges). For group comparisons: Categorical variables were analyzed using the Fisher’s exact test or the Pearson Chi-square test. Continuous variables were compared via one-way analysis of variance (ANOVA) (for normally distributed data) or appropriate nonparametric tests (for skewed data). Survival outcomes were analyzed using the Kaplan-Meier method, with the Breslow test used to compare survival curves across groups. Landmark analysis was further performed to stratify survival by a critical time point (600 days post-diagnosis, ≈20 months), which was selected based on previous anti-GBM disease studies reporting that ~80% of adverse events (including mortality) occur within 18–24 months post-diagnosis (15). Besides, the Kaplan-Meier survival curves in this study showed a clear separation trend among the three groups at 600 days. To ensure statistical robustness, follow-up data were censored at this time point, and the hazard ratios (HRs) among the three groups were quantified. Similar trends were subsequently observed at multiple adjacent time points (700 and 800 days). Given the study’s focus on the mechanisms underlying early event occurrence, 600 days was selected as the landmark cutoff. Therefore the survival curve was split into two intervals (0–600 days and >600 days), and group differences were tested separately for each interval. Cox proportional hazards model was used to adjust for potential confounders. All statistical analyses were conducted using IBM SPSS Statistics for Windows (version 27.0) and R software (version 4.5.2). A two-sided P < 0.05 was considered statistically significant.

## 3 Results

### 3.1 Demographic and clinical data

A total of 67 patients with anti-GBM disease were enrolled in this study, with 30 assigned to group 1 (classic anti-GBM group), 18 to group 2(anti-GBM-ANCA double-positive group), and 19 to group 3 (anti-GBM-non-ANCA autoantibody overlap group). Baseline demographic and clinical characteristics are summarized in **Table 2**. Although group 2 had the highest proportion of male patients (61.11%), this difference was not statistically significant across the three groups. Notably, the age of onset in group 2 was significantly higher than that in groups 1 and 3 (p < 0.001). With respect to clinical symptoms, no significant intergroup differences were observed in the prevalence of preceding infection, pulmonary involvement, abnormal blood pressure, gross hematuria, or oliguria/anuria (**Table 2**).

**Table 2:** Clinical characteristics of three different groups of patients with anti-GBM disease.

	Group1 (n=30)	Group2 (n=18)	Group3 (n=19)	P-value
Male (n, %)	13 (43.33%)	11 (61.11%)	8 (42.11%)	0.414
Age of onset (years)	54.57 ± 18.80	69.89 ± 7.85	49.42 ± 16.20	<0.001
Smoking history (n, %)	9 (30.00%)	10 (55.56%)	5 (26.32%)	0.120
visible hematuria (n, %)	20 (66.67%)	10 (55.56%)	14 (73.68%)	0.513
Oliguria/anuria (n, %)	20 (66.67%)	13 (72.22%)	11 (57.89%)	0.649
Preceding infection (n, %)	15 (50.00%)	9 (50.00%)	8 (42.11%)	0.844
Pulmonary (n, %) involvement (n, %)	1 (3.33%)	3 (16.67%)	4 (21.05%)	0.133
Hypertension (n, %)	17 (56.67%)	9 (50.00%)	11 (57.89%)	0.870
Systolic pressure (mmHg)	148.30 ± 19.14	137.44 ± 18.98	140.47 ± 18.78	0.130
Diastolic pressure (mmHg)	83.53 ± 14.28	80.56 ± 12.22	82.63 ± 12.54	0.440
Leukocyte (10^9^/L)	9.49 ± 4.15	9.56 ± 2.93	9.69 ± 3.05	0.982
Granulocyte (10^9^/L)	7.66 ± 4.12	7.55 ± 2.95	7.70 ± 3.13	0.991
Lymphocyte (10^9^/L)	0.91 (0.68,1.19)	1.05 (0.77,1.64)	1.05 (0.65,1.48)	0.730
Hemoglobin (g/L)	90.50 (79.75,102.25)	77.00 (71.75,91.25)	91.00 (82.50,103.50)	0.064
Platelet (10^9^/L)	218.00 (174.25,283.25)	232.00 (167.25,298.75)	192.00 (136.50,276.00)	0.805
Serum creatinine (umol/L)	627.59 ± 373.53	551.57 ± 237.01	662.88 ± 361.61	0.595
Serum urea (mmol/L)	21.81 ± 10.69	17.43 ± 6.82	22.72 ± 10.58	0.212
eGFR (mL/min/1.73m²)	7.25 (4.45,13.05)	7.15 (5.50,13.37)	6.60 (5,10,11.35)	0.914
Serum total protein (g/L)	56.56 ± 9.97	59.18 ± 5.85	58.32 ± 10.08	0.599
Serum albumin (g/L)	28.57 ± 6.68	27.58 ± 4.93	27.97 ± 5.39	0.844
Serum globulin (g/L)	27.99 ± 6.43	31.61 ± 6.20	29.57 ± 7.59	0.202
Total cholesterol (mmol/L)	4.09 (3.80,5.52)	3.88 (3.19,4.54)	4.07 (3.61,4.96)	0.351
ALT (U/L)	11.75 (9.53,21.28)	9.45 (7.35,15.77)	14.80 (7.30,18.75)	0.369
AST (U/L)	17.85 (14.83,22.57)	16.40 (15.27,19.77)	19.40 (13.50,30.70)	0.778
AKP (U/L)	82.50 (71.20,112.00)	82.10 (65.03,99.75)	76.00 (67.00,102.00)	0.862
LDH (U/L)	258.50 (213.25,312.00)	224.00 (165.50,290.25)	313.00 (224.50,378.00)	0.076
C reactive protein (mg/L)	50.85 (7.70,128.75)	62.15 (8.35,96.42)	55.90 (18.70,112.50)	0.926
Complement 3 (g/L)	0.93 ± 0.26	0.85 ± 0.19	0.91 ± 0.33	0.638
Complement 4 (g/L)	0.24 (0.22,0.29)	0.25 (0.22,0.30)	0.25 (0.20,0.37)	0.740
Anti-GBM antibody titer (IU/mL)	141.20 (102.90,257.70)	104.25 (52.15,137.07)	181.70 (121.05,555.40)	0.044
IgG4 positive	0 (0.00)	6 (42.86)	2 (11.76)	<0.001
Serum IgG4 levels (g/L)	0.29 (0.10,0.53)	1.55 (0.65,5.71)	0.43 (0.26,0.63)	<0.001
Urinary protein (g/24h)	2.20 (0.84,5.81)	0.69 (0.49,1.40)	2.22 (1,27.3.19)	0.064

ALT, Alanine Aminotransferase; AST, Aspartate Aminotransferase; AKP, Alkaline Phosphatase; LDH, Lactate Dehydrogenase; eGFR, estimated Glomerular Filtration Rate. The eGFR is calculated by the CKD-EPI formula.

Further analysis of baseline laboratory data revealed no significant intergroup differences in complete blood count or renal function parameters, including serum creatinine, urea, and estimated glomerular filtration rate (eGFR). However, a notable difference emerged in anti-GBM antibody titers at diagnosis, group 3 had the highest median titer, followed by group 1, and this intergroup variation was statistically significant [median (interquartile range): group 1 104.25(52.15-137.07); group 2 141.20 (102.90-257.70); group 3 181.70(121.05-555.40); p=0.044]. The IgG4 positive rate (p < 0.001) and serum IgG4 levels differed significantly among the three groups, with group 2 showing the highest and group 1 the lowest values [median (interquartile range): group 1 0.29(0.10-0.53); group 2 1.55(0.65-5.71); group 3 0.43(0.26-0.63); p < 0.001] (**Table 2**).

### 3.2 Renal pathological characteristics

Among the enrolled patients, 25 underwent renal biopsy,and no significant intergroup differences were observed in renal biopsy pathological features (**Table 3**). Specifically, the three groups showed comparable distributions in terms of glomerular crescent formation rate, glomerular capillary loop fibrinoid necrosis, Bowman’s capsule rupture, and the deposition patterns of immunoglobulins (IgG, IgA, IgM), complements (C3, C4), and fibrinogen; indices of chronic renal lesions (e.g., glomerulosclerosis, tubulointerstitial fibrosis, arterial hyalinosis) also did not differ statistically across groups.

**Table 3:** Comparison of pathological characteristics of renal biopsy among the three groups of patients.

	Total (n=25)	Group 1 (n=11)	Group 2 (n=5)	Group 3 (n=9)	P-value
Light microscope					
Total no. of glomeruli	16.27 ± 8.08	14.91 ± 8.49	18.00 ± 8.09	16.90 ± 8.24	0.756
Capillary loops necrosis, n (%)	5 (20.00)	4 (28.57)	0 (0.00)	1 (16.67)	0.214
Crescents, n (%)	21 (84.00)	9 (81.82)	4 (80.00)	8 (88.89)	1.000
Crescents	13.20 ± 9.30	11.55 ± 9.62	13.40 ± 9.91	15.11 ± 9.32	0.712
Cellular crescent, n (%)	21 (84.00)	9 (81.82)	4 (80.00)	8 (88.89)	1.000
Cellular crescents	7.00 (3.00, 17.00)	3.00 (1.50,11.50)	6.00 (4.00, 11.00)	15.00 (7.00,18.00)	0.282
Fibro cellular crescents, n (%)	10 (40.00)	4 (36.36)	4 (80.00)	2 (22.22)	0.165
Fibro cellular crescents	0.00 (0.00, 4.00)	0.00 (0.00,4.00)	2.00 (1.00, 4.00)	0.00 (0.00, 0.00)	0.325
Fibrous crescents, n (%)	9 (36.00)	3 (27.27)	3 (60.00)	3 (33.33)	0.484
Fibrous crescents	0.00 (0.00, 1.00)	0.00 (0.00, 1.00)	1.00 (0.00, 1.00)	0.00 (0.00, 1.00)	0.681
Renal tubular necrosis, n (%)	9 (36.00)	4 (28.57)	1 (20.00)	4 (66.67)	0.276
Renal tubular atrophy, n (%)	12 (48.00)	7 (50.00)	3 (60.00)	2 (33.33)	0.753
Immunofluorescence					
IgA, n (%)	5 (20.00)	2 (18.18)	1 (20.00)	2 (22.22)	1.000
IgG, n (%)	21 (84.00)	8 (72.73)	4 (80.00)	9 (100.00)	0.223
IgM, n (%)	12 (48.00)	4 (36.36)	5 (100.00)	3 (33.33)	0.044
C3, n (%)	19 (76.00)	8 (72.73)	4 (80.00)	7 (77.78)	1.000
C4, n (%)	2 (8.00)	0 (0.00)	1 (20.00)	1 (11.11)	0.303
C1q, n (%)	8 (32.00)	3 (27.27)	2 (40.00)	3 (33.33)	1.000
Fibrin, n (%)	23 (92.00)	10 (90.91)	5 (100.00)	8 (88.89)	1.000
IgG1, n (%)	18 (72.00)	6 (54.55)	3 (60.00)	9 (100.00)	0.051
IgG2, n (%)	7 (28.00)	1 (9.09)	1 (20.00)	5 (55.56)	0.080
IgG3, n (%)	8 (32.00)	3 (27.27)	2 (40.00)	3 (33.33)	1.000
IgG4, n (%)	14 (56.00)	6 (54.55)	4 (80.00)	4 (44.44)	0.509

### 3.3 Treatment and follow-up clinical outcomes

All enrolled patients received standard treatment regimens for anti-GBM disease, including plasma exchange, glucocorticoid pulse therapy, cyclophosphamide-based immunosuppression, and/or rituximab. Statistical analysis showed no significant intergroup differences in the distribution of treatment modalities (**Table 4**).

**Table 4:** Comparison of treatment modalities and outcome among three groups of anti-GBM disease patients.

Medication use	Group 1	Group 2	Group 3	P value
GC	28 (93.33%)	16 (88.99%)	16 (84.21%)	0.608
CTX	16 (53.33%)	11 (61.11%)	10 (52.63%)	0.841
RTX	4 (13.79%)	4 (22.22%)	2 (10.53%)	0.682
ACEI/ARB	3 (10.00%)	1 (5.56%)	3 (15.79%)	0.689
Plasmapheresis	12 (40.00%)	9 (50.00%)	14 (73.68%)	0.069
Initial KRT	21 (70.00%)	12 (66.67%)	16 (84.21%)	0.423
Follow up KRT	9 (30.00%)	6 (33.3%)	5 (26.32%)	0.897
Death	3 (10.00%)	6 (33.3%)	4 (21.05%)	0.133

GC, glucocorticoid; CTX, cyclophosphamide; RTX, Rituximab; KRT, Kidney Replacement Therapy.

During follow-up (until January 2024), there were no statistically significant differences among the three groups in two key renal-related outcomes: (1) the proportion of patients requiring hemodialysis initiation at disease onset; (2) the proportion of patients remaining dependent on hemodialysis (including those who died) by the end of the follow-up period. Additionally, no significant variation in all-cause mortality was observed across the three groups at the conclusion of follow-up.

### 3.4 Differences in early survival among anti-GBM patients with distinct immunological overlap profiles

**Figure 2:**
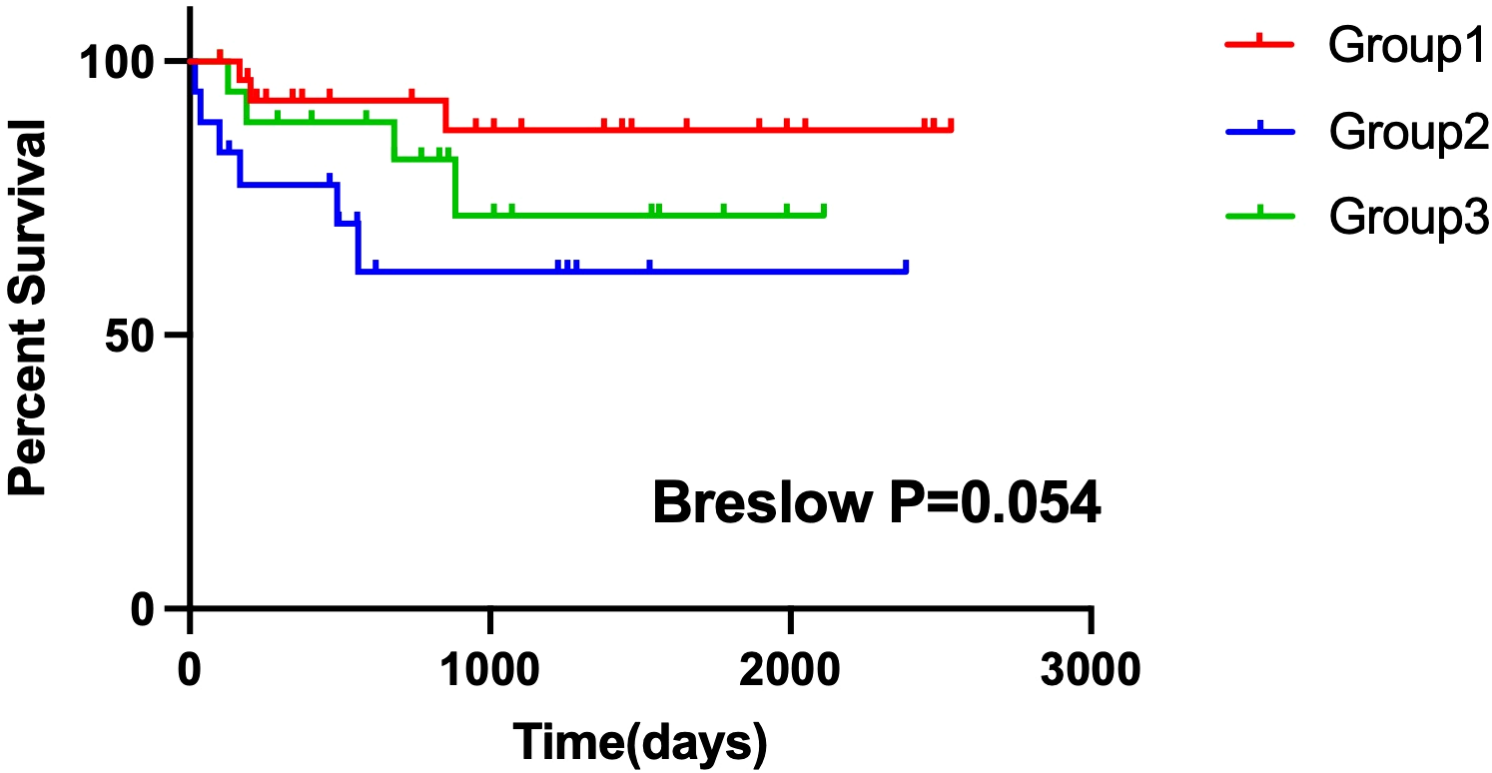
Kaplan–Meier’s survival curve for three groups of anti-GBM-GN patients. Kaplan–Meier’s survival curve across the three patient groups showed no significant intergroup difference in overall survival (Breslow test, p = 0.054).

**Figure 3:**
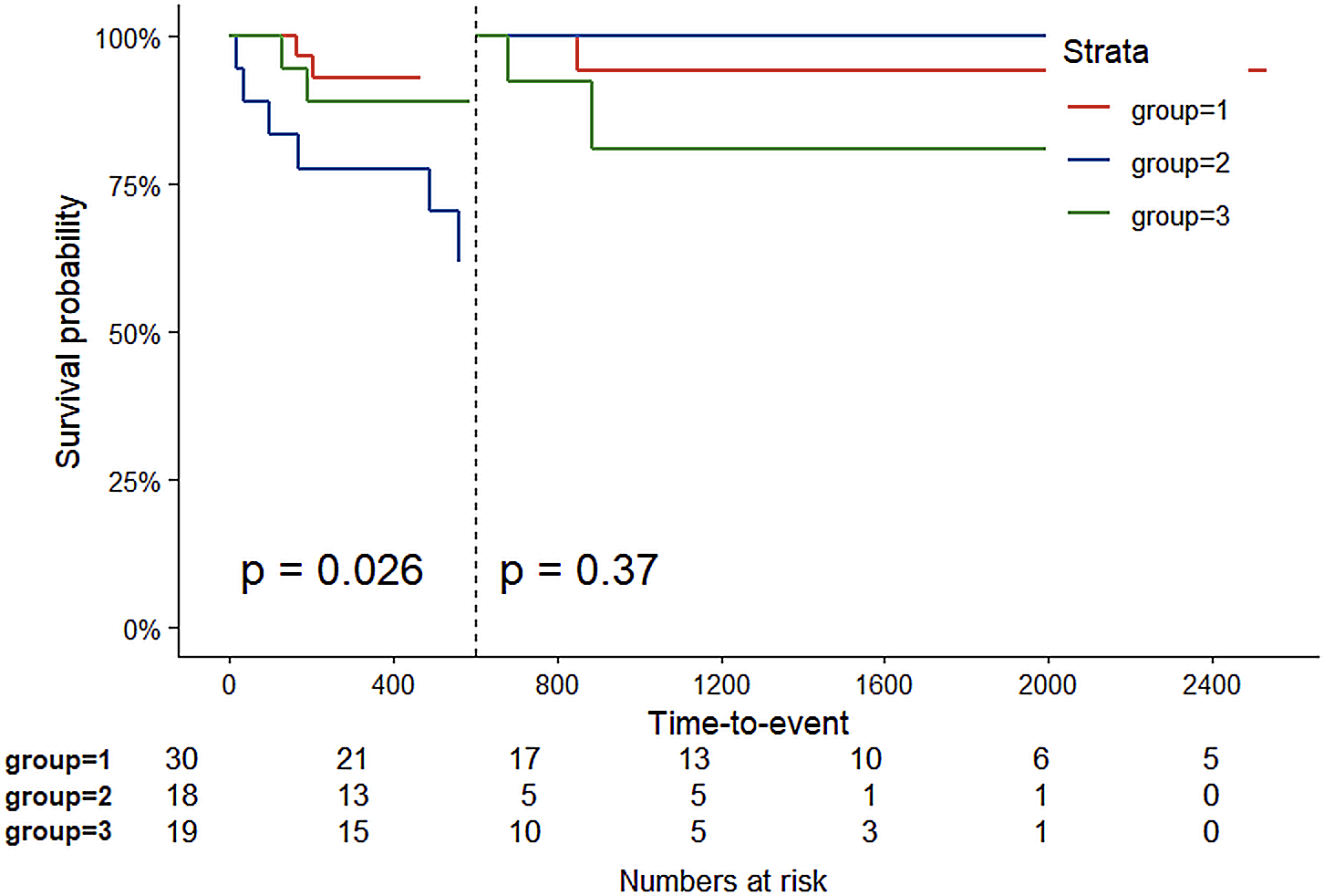
Landmark analyses (0 to 600 and after 600) for three groups of anti-GBM-GN patients. Landmark analysis showed the statistically significant intergroup survival difference before the 600-day landmark was (p = 0.026) and no significant survival difference after the 600-day landmark (p = 0.370).

**Figure 4:**
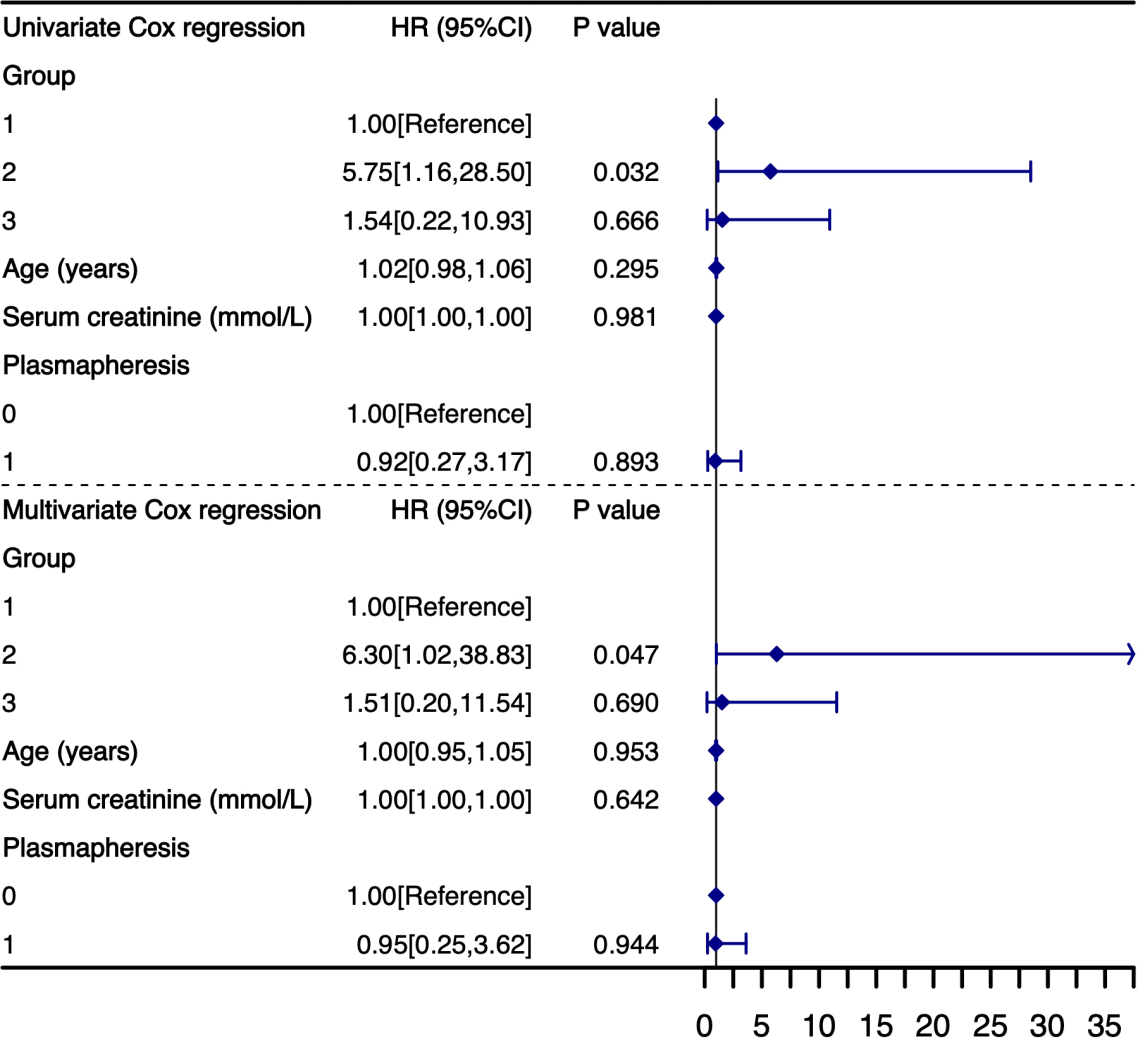
Cox proportional hazards model of early survival for three groups of anti-GBM-GN patients. Univariate Cox proportional hazards regression analysis was employed to identify risk factors associated with early survival in the three groups of anti-GBM-GN patients. Multivariate Cox regression analysis was used to adjust for potential confounders, and showed that the anti-GBM-ANCA double-positive group (group 2) had the higher death risk compared to group 1 (HR = 6.30, 95% CI = 1.02~38.83, p =0.047).

With January 2024 as the follow-up endpoint, we further analyzed survival differences across the three patient groups (**Figure 2**). Although no significant intergroup difference in overall survival was observed, a trend toward variation in early survival rates was noted (Breslow test, p = 0.054).To clarify this trend, we performed a landmark analysis using 600 days post-diagnosis as the critical time point. The results showed that before 600 days, the anti-GBM-ANCA double-positive group (group 2) had the lowest survival rate, while the anti-GBM-non-ANCA autoantibody overlap group (group 3) exhibited moderately better survival; the classic anti-GBM group (group 1) had the most favorable early survival. This intergroup difference in survival before the 600-day landmark was statistically significant (p = 0.026). In contrast, no significant survival differences were detected among the three groups after the 600-day landmark (p = 0.370) (**Figure 3**). The Cox proportional hazards model showed that the anti-GBM-ANCA double-positive group (group 2) had the higher death risk compared to group 1 (HR = 6.30, 95% CI = 1.02~38.83, p =0.047) after adjusting for age, plasmapheresis, and serum creatinine in early 600 days (**Figure 4**).

## 4 Discussion

Anti-GBM disease is an autoimmune nephropathy with a complex pathogenesis. Since Goodpasture first described “pulmonary-renal syndrome” in 1919 and localized its target to the glomerular basement membrane (16, 17), and the α3(IV)NC1 was confirmed as the sole autoantigen in the 1990s, anti-GBM disease has long been regarded as a classic model of “single antibody–linear deposition–complement activation.” With advances in research, the current understanding of this disease has evolved toward a complex paradigm of “multi-antigen, multi-antibody, multi-pathway”. The discovery of novel target antigens, such as the α4 and α5 chains of type IV collagen and the laminin-521, has expanded the antigenic spectrum (18, 19). The identification of IgG subclasses (e.g., IgG4) has enriched the diversity of pathogenic antibody types. Furthermore, several clinical reports have documented the co-occurrence of anti-GBM antibodies with MPO-ANCA, phospholipase A2 receptor (PLA2R) antibodies, and anti-double-stranded DNA (anti-dsDNA) antibodies leading to the proposal of “immunologic overlap” (7).Due to the rarity of anti-GBM disease, previous studies on anti-GBM disease with concurrent antibodies have been limited to sporadic case reports, lacking systematic prognostic comparisons. Therefore, this study proposes the concept of “overlap-immune anti-GBM syndrome”. By comparing the clinical and prognostic characteristics of different immunologically overlapping subtypes of anti-basement membrane disease, this research aims to deepen the understanding of the disease, facilitate further elucidation of its pathogenic mechanisms, enable the identification of distinct disease subtypes, improve prognostic prediction accuracy, and provide implications for clinical treatment strategies.

In our cohort, patients with classic anti-GBM disease, GBM–ANCA double-positive disease, and Anti-GBM-non-ANCA autoantibody overlap disease were analyzed separately. At baseline, anti-GBM antibody titers were significantly lower in the ANCA-positive subgroups, consistent with the observations reported by McAdoo et al (20). Previous studies have indicated that this discrepancy reflects epitope spreading: compared with anti-GBM single-positive individuals, double-positive patients harbor a broader spectrum of anti-GBM antibodies and exhibit reduced reactivity against the α3(IV)NC1 domain (21). Consistent with this, the deposition of IgM antibodies in the kidneys of double positive patients is also more significant. Additionally, ANCA-driven vasculitis induces early basement membrane injury, which may lead to prolonged exposure of cryptic collagen epitopes and facilitate the rapid formation of *in-situ* immune-complex. Circulating free anti-GBM antibodies are thereby consumed. Consequently, a substantial proportion of antibodies may not be detected by routine clinical assays (22), resulting in a false impression of low antibody titers. Notably, the resulting laboratory profile of “low-antibody levels” often does not align with the severe organ damage observed clinically (23).

Compared to patients with isolated anti-GBM disease, those with overlap-immune anti-GBM syndromes (excluding ANCA-associated cases) tend to have an earlier disease onset and higher serum anti-GBM antibodies titers. This suggests that the coexistence of multiple autoantibodies may generate synergistically amplified immune signals and confer a more intense immunophenotype. The temporal sequence underlying the emergence of anti-GBM antibodies relative to other autoantibodies remains unclear. Some studies propose that anti-GBM antibodies often develop secondarily to ANCA, anti-PLA2R, or other antibodies: the initial damage to the basement membrane induced by these antecedent antibodies cryptic α3(IV)NC1 epitopes, which subsequently triggers the production of anti-GBM antibody (24). However, the opposite sequence has also been reported (25). Regardless of chronological order, we speculate that the aggregated autoantibody pool may constitute an “expanded immune cluster” that continuously drives epitope spreading and diversification of immune deposits during disease progression.

In the present cohort, Kaplan–Meier survival curves revealed that overall mortality rates converged across the three groups beyond 600 days, whereas significant separation occurred within the early phase (≤600 days): patients with double positivity (anti-GBM + ANCA) carried the highest early mortality risk, followed by those with anti-GBM plus non-ANCA antibodies, while individuals with isolated anti-GBM disease had the lowest risk. The poor prognosis observed in the double-positive subset is consistent with findings from previous anti-GBM + ANCA cohorts (20, 26). The poorer early survival observed in the ANCA-overlap cohort may reflect age, comorbidity burden, vasculitic phenotype, and differential treatment responsiveness. This may also be attributed to the superimposition effect of two distinct immune pathways: linear IgG deposition along the glomerular basement membrane mediated by anti-GBM antibodies, and widespread capillary disruption, pulmonary hemorrhage, and rapidly progressive glomerulonephritis induced by ANCA-driven vasculitis. Together, these two pathways constitute a “dual immune hit” that exponentially amplifies tissue injury, which explains why this subtype has the worst early prognosis among the three groups.

In contrast, although patients with anti-GBM combined with non-ANCA autoantibodies do not exhibit the fulminant vasculitis manifestations seen in ANCA-positive cases, their early mortality risk is still approximately twice that of patients with isolated anti-GBM disease. We hypothesize that this intermediate risk may stems from epitope spreading (27). Tissue injury or chronic inflammation exposes previously sequestered self−epitopes; these newly revealed determinants are internalized by antigen−presenting cells, processed, and presented to T cells, which in turn activate B cells specific for the novel epitopes (28, 29). The outcome is a broader, polyclonal autoimmune response that can augment disease severity and drive the emergence of secondary autoantibodies. In our study, this expansion may lead to the formation of granular immune complexes that deposit along the outer aspect of the basement membrane. The coexistence of granular and linear IgG deposits results in a mixed pattern of glomerular injury. Given that these additional epitopes are relatively benign and predominantly induce IgG2 or IgG4-mediated immune responses—an IgG subclass with weak complement-fixing capacity—the intensity of complement activation (e.g.,C5a generation, membrane attack complex [MAC] formation) is lower than that in ANCA-mediated vasculitis (6). Consequently, although the inflammatory burden is higher than in isolated anti-GBM disease, it remains lower than in the double-positive group. This limits direct capillary disruption, thereby resulting in an intermediate prognosis, which is consistent with the survival curve findings in our cohort.

Collectively, in overlap-immune anti-GBM syndrome characterized by an expanded antibody repertoire, anti-GBM antibody titers alone can no longer accurately predict prognosis. Clinicians should not underestimate the severity of overlap-immune anti-GBM syndrome. Early immunological stratification via comprehensive antibody screening, followed by individualized therapeutic strategies tailored to the extent of epitope spreading and the characteristics of immune deposits, is essential to capitalize on the early-to-mid-stage therapeutic window. Dynamic monitoring of multiple immune parameters during follow-up will facilitate early and precise intervention in high-risk patients, thereby improving clinical outcomes.

However, our study also has the following limitations. First, due to the inherent rarity of anti-GBM disease, the sample size was limited. We could only perform preliminary grouping based on the presence or absence of other autoantibodies and were unable to conduct detailed subgroup analyses of different antibody combinations. The anti-GBM-non-ANCA overlap cohort exhibits distinct heterogeneity owing to highly heterogeneous antibody profiles. If future research can include more patients and conduct detailed subgroup analysis of different antibody combinations, it will be beneficial for accurately assessing the risk differences and clinical significance among various complex immune phenotypes. Second, the adjustment for cardiovascular and pulmonary confounders is insufficient. Although there were no statistical differences in the smoking history and pulmonary involvement rate among groups, numerical differences remain noteworthy. Conditions discrepancy such as coronary artery disease, chronic obstructive pulmonary disease, smoking-related pulmonary injury, and respiratory infection may have confounded, to some extent, the true contribution of immunological factors to the study outcomes. Future large-sample studies may include more confounding factors for adjustment to obtain more robust conclusion. Third, this study is retrospective, single-center, and based primarily on serological subgrouping without mechanistic immunologic assays. The mechanistic explanations regarding epitope spreading, immune cluster expansion, complement activation differences, and mixed immune-deposit biology remain speculative.

The present study identified distinct subgroups of anti-GBM disease. Future studies should expand the scale of multicenter sample cohorts to enable adequate subgroup comparisons across different antibody combinations. Additionally, high-resolution imaging from transmission electron microscopy and laser microdissection techniques should be integrated to systematically evaluate glomerular basement membrane epitope exposure, slit diaphragm formation and immune-complex deposition patterns. Such an approach will facilitate more refined risk stratification and provide a more reliable basis for the individualized treatment of overlap-immune anti-GBM syndrome.

## Data Availability

The raw data supporting the conclusions of this article will be made available by the authors, without undue reservation.
